# Prevalence and treatment of vitamin K deficiency in paediatric patients with recessive dystrophic epidermolysis bullosa‐severe subtype

**DOI:** 10.1002/ski2.14

**Published:** 2021-03-31

**Authors:** N. Yerlett, G. Petrof, K. Holsgrove, A. Martinez

**Affiliations:** ^1^ Department of Dermatology Great Ormond Street Hospital for Children London UK

## Abstract

**Introduction:**

Patients with recessive dystrophic epidermolysis bullosa‐severe subtype (RDEB‐S) are at risk of vitamin K deficiency, potentially causing abnormal clotting, excessive bleeding, poor bone metabolism and abnormal vascular calcification. This study quantifies vitamin K deficiency prevalence in this cohort and identifies potential risk‐factors to prevent deficiency.

**Methods:**

Patients with RDEB‐S who attended the EB service between 2014 and 2020 were included. Serum vitamin K and PIVKAII were measured as part of the usual nutritional blood screen. Dietetic and medical notes were reviewed to establish: antibiotic use, enteral feed intake and micronutrient supplementation.

**Results:**

A total of 16/25 64% (10/16 female), of children aged 22–180 months, had serum vitamin K and PIVKAII analysed. Six of sixteen (37.5%) patients had vitamin K deficiency requiring supplementation. Two of six (33.3%) normalized serum vitamin K after 12 weeks supplementation with oral menadiol diphosphate. Four of six (66.6%) await retesting following supplementation. Six of six (100%) patients with vitamin K deficiency were not consuming a gastrostomy/sip feed. Nine of ten (90%) patients with sufficient vitamin K levels were consuming either; more than 200 ml prescribed sip feed or more than 400–800 ml gastrostomy feed daily (containing 5.9–11 µg/100 ml vitamin K). Patients who were consuming either more than 200 ml prescribed sip feed or more than 400–800 ml gastrostomy feed daily (containing 5.9–11 µg/100 ml vitamin K) were significantly less likely to suffer from vitamin K deficiency (0.08 odds ratio [(1/7)/(5/3)] with significance level *p* = 0.0342 [95% CI: 0.0074–0.8275]). Sixteen of sixteen (100%) received antibiotics (range 0–4 courses/year; median, 3; IQR, 3). Patients with the most frequent antibiotics (*n* = 4) had normal vitamin K and PIVKAII levels if they consumed a minimum of 200 ml prescribed sip feed or 400–800 ml gastrostomy feed daily. Sixteen of sixteen (100%) patients took a multivitamin/mineral supplement; none contained vitamin K.

**Summary:**

The prevalence of vitamin K deficiency is 37.5% in this cohort. Patients whom were not consuming gastrostomy/sip feeds of at least 200 ml daily were at greatest risk of vitamin K deficiency. Patients on a micronutrient supplement remain at risk of vitamin K deficiency, as most contain no vitamin K. Prescribing a vitamin/mineral supplement that contains vitamin K is recommended. Twelve‐week supplementation of oral vitamin K (5 mg/day for 1–10 years and 10 mg/day for 12–17 years) adequately improved stores.

## Graphical abstract

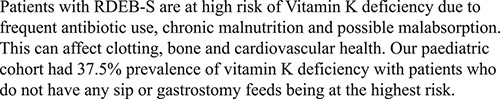

1

**What is already known about this topic?**

- Vitamin K is rarely tested in paediatric patients.
- Patients with RDEB‐S are at risk of Vitamin K deficiency due to frequent antibiotic use, chronic malnutrition and gastrointestinal malabsorption.

**What does this study add?**

- The prevalence of Vitamin K deficiency is high in patients with RDEB‐S.
- Serum vitamin K and PIVKA‐II should be tested at least annually in at‐risk patients.
- Treating vitamin K deficiency is easy to achieve and can prevent secondary complications associated with clotting and bone health.
- Patients with RDEB‐S who consume <200mls of an enteral feed should be prescribed a general micronutrient supplement containing vitamin K to prevent deficiency.

## 2 INTRODUCTION

Epidermolysis bullosa (EB) comprises of a group of rare genetically determined skin blistering disorders characterized by extreme fragility of the skin and all mucous membranes. All subtypes result from genetic mutations which cause a defect in, or a partial/full absence of, structural proteins in the dermal–epidermal basement membrane.^1^, ^2^ The resultant reduced, or absent, adhesion means that any knocks or friction can cause the skin to blister. Patients can have life‐long chronic wounds, which are at constant risk of infection and bleeding.^2^ RDEB‐S is one of the most severe subtypes of EB with patients often having total absence of the structural protein Collagen VII.^1^, ^2^

Sufficient nutritional intake can often be a challenge due to oral mucosa damage, poor dentition, microstomia, oesophageal stricturing and reduced tongue movement in patients with RDEB‐S.^2^ These patients have very high nutritional requirements of macro and micronutrients due to constant wound healing. The background level of chronic inflammation^2^ affects many internal pathways, in particular the growth axis, leading to chronic and severe faltering growth from an early age which requires intensive dietetic management to attempt to remedy.^2^

A number of mechanisms are thought to contribute to the observed low bone mineral density (BMD) for chronological age seen in RDEB‐S.^3^, ^4^, ^5^ These include reduced mobility, poor nutritional intake, reduced sunlight exposure (from restricted outdoor activities and extensive bandaging), chronic inflammation (proinflammatory cytokines increase osteoclastic activity) and pubertal delay.^3^, ^4^, ^5^ In addition, gastrointestinal complications may affect absorption of relevant nutrients.^6^

### 2.1 Vitamin K

Vitamin K refers to a family of compounds with a common chemical structure of 2‐methyl‐1,4‐naphthoquinone.^7^ Vitamin K is an essential fat‐soluble vitamin.

Vitamin K is required for the γ‐carboxylation of specific glutamic acid residues within the Gla domain of at least 17 vitamin‐k‐dependent proteins.^7^, ^8^, ^9^

Functions of vitamin K are historically associated with blood coagulation; specifically the production of clotting factors, II, VII, IX and X.^8^, ^10^ Without sufficient levels of vitamin K, a patient can be at risk of insufficient clotting and excessive bleeding.^8^, ^10^, ^11^ In addition, more recent evidence has shown that the vitamin K‐dependent proteins are also involved in bone metabolism and vascular calcification: specifically with the γ‐carboxylation of osteocalcin (bone building), periostin may promote modification of osteoblasts (bone modelling) and prevent the calcification of vessel walls.^8^, ^10^, ^12^ ^,^ ^13^ There is also some evidence of a synergistic relationship between vitamin D and vitamin K to maintain serum Calcium levels.^10^

The main two forms of vitamin K comprise of the following.

(1) K_1_ (phylloquinone)—sourced from mainly green leafy veg, such as broccoli and spinach, some vegetable oils and some cereal grains. Smaller amounts are also found in meat and dairy foods.
(2) K_2_ (menaquinones)—a collection of varying isoprenologues produced by gastrointestinal bacteria (especially in the ileum), some of which is also converted from phylloquinone.^11^ Sources may also include: dairy foods, especially when fermented.^14^

Liver stores of K_1_ are relatively small (10%–15%) and a large proportion is lost to excretion; therefore, turnover is high and a constant supply is required to maintain serum vitamin K levels.^10^

Dietary analysis of vitamin K can be inadequate due to the lack of specificity of the amounts of the varying forms of vitamin K present in foods.^7^

### 2.2 Serum testing for vitamin K

Vitamin K serum testing is not common in paediatric medicine. Commonly, coagulation assays such as the prothrombin time have been used as indicators of clotting and vitamin K status^8^; however, coagulation is not the only function of vitamin K and so this does not help the clinician determine a nutritional vitamin K deficiency per se as they lack full sensitivity and specificity to do that.

The measurement of serum phylloquinone (K_1_) is the most commonly used marker of vitamin K status and reflects the current serum concentration. Concentrations of less than 0.15 μg/L are suggestive of a deficiency. This value alone, however, cannot give serum status on the other vitamin K homologues and it must be noted that this K_1_ level may be effected by very recent dietary intake.^8^, ^10^

A second serum marker can be tested simultaneously to give a more accurate picture of abundance and utilization of vitamin K; the protein induced by vitamin K absence/antagonism (PIVKA‐II). PIVKA‐II serum level can give a retrospective look at the hepatic vitamin K status. PIVKA‐II indicates whether marginal tissue stores have reached a point at which the hepatic ɣ‐carboxylation of factor II has been compromised^8^; that is, if PIVKA‐II is raised above the reference range, this may indicate subclinical or historic vitamin K deficiency. In our cohort, both serum K_1_ and serum PIVKAII were measured to maximize accuracy and understanding of the full clinical picture.

### 2.3 Potential vitamin K deficiency in RDEB‐S

Patients with severe types of EB are theoretically at high risk of suffering from vitamin K deficiency due to their reduced intake of foods containing vitamin K. Patients with RDEB‐S may have frequent infections and require prescription of antibiotics, in addition to suffering gastrointestinal malabsorption and severe chronic constipation.^6^ Not only does frequent antibiotic use cause potential for gastrointestinal dysbiosis, slow transit time has also been shown to disrupt the gastrointestinal microbiome.^15^ These factors causing high risk of dysbiosis may potentially reduce the number of gastrointestinal bacteria that produce vitamin K_2_.^11^

Jenkins et al.^9^ found that 91% of a paediatric burns population were found to have vitamin k deficiency which was statistically significantly affected by: antibiotic use, percent of surface area of skin effected and number of days with diarrhoea. Although not directly relatable, the chronic nature of EB wounds and frequent antibiotic use can draw direct comparisons.

Patients with RDEB‐S often suffer from other complications secondary to vitamin K deficiency; osteopenia, osteoporosis, fractures, prolonged bleeding exacerbating chronic iron‐deficiency anaemia.^2^, ^3^, ^4^, ^5^ It can be hypothesized that in patients with RDEB‐S, testing for and treating vitamin K deficiency may play a role in reducing the frequency and severity of these common secondary complications.

## 3 METHODS

Patients with RDEB who attended the EB specialist service at Great Ormond Street Hospital for full review by the multidisciplinary team between 2014 and 2020 were included in the audit. Routine practice is to take extended nutritional blood tests annually (or more frequently if indicated); therefore, this was carried out as usual and included serum vitamin K and serum PIVKAII requests. Samples were sent to Guy's and St. Thomas' Laboratory as per normal protocol for the specific assays. The EB specialist dietitian collated results once available on the electronic patient record. The results were discussed with the wider MDT (including EB clinical nurse specialist and consultant paediatric dermatologist) and a treatment plan was agreed. The EB specialist dietitian subsequently informed the families. Repeat serum samples were taken at the next usual full blood review.

## 4 RESULTS

Twenty‐five paediatric patients with RDEB‐S were reviewed between January 2014 and June 2020 within the tertiary EB service and had nutritional bloods taken as part of a routine EB review. Sixteen out of twenty‐five, 64% (10/16 female), age range 22–180 months, successfully had their vitamin K and PIVKA II status checked.

Seven out of twenty‐five (28%) patients had incomplete testing due to the blood request form not being completed or the blood sample not being received by the laboratory. Two out of twenty‐five (8%) patients had bloods sent for vitamin K analysis but the volume of the sample was not sufficient to be assayed.

One out of sixteen (6.25%) patients has continuing high PIVKAII results (three samples sent over the study period) although vitamin K is within the reference range.

Of the twenty‐five patients who successfully had vitamin K status checked, 6/16 (37.5%) patients had vitamin K deficiency requiring a course of oral supplementation (Table 1).

Sixteen out of twenty‐five patients (100%) received a course of prescribed antibiotics during the observation period (range 0–4 courses prescribed [median = 3; IQR = 3] per annum). Patients with the greatest number of courses of antibiotics (*n* = 4) were still protected from vitamin K deficiency if they were consuming a minimum of either; 200 ml of a nutritionally complete sip feed or 400–800 ml a gastrostomy feed every day; containing vitamin K (content 5.9–11 µg/100 ml).

Two out of 6 (33.3%) successfully showed normal results after a short course of supplementation. The remaining 4/6 (66.7%) have not yet had a successful re‐test of the level following their supplementation.

Six out of six (100%) of the patients that had demonstrated vitamin K deficiency were not on a gastrostomy or sip feed that contains vitamin K. Nine out of ten (90%) patients that had sufficient levels were on at least one nutritional sip feed (200 ml minimum) per day or a gastrostomy feed every day (ranged from 400–800 ml); all containing vitamin K (Table 1). The 1/10 (10%) patient that had normal serum vitamin K and no sip feeds did have repeated raised PIVKAII levels; this patient does have a diet very rich in vitamin K. Patients who were consuming either more than 200 ml prescribed sip feed or more than 400–800 ml gastrostomy feed daily (containing 5.9–11 µg/100 ml vitamin K) were significantly less likely to suffer from vitamin K deficiency (0.08 odds ratio [(1/7)/(5/3)] with significance level *p* = 0.0342 (95% CI: 0.0074–0.8275)).

All 16 patients were on a multivitamin and mineral supplement; however, it is uncommon for paediatric supplements to contain vitamin K, so none of the patients in the study had a multivitamin and mineral supplement containing vitamin K  .

**TABLE 1:** Serum levels of vitamin K and PIVKA II, antibiotic frequency and enteral feed intake in patients with RDEB‐S

Patient	Age at first visit K test (months)	Sex	PIVKAII ‐first test (mAU/ml)	Reference range (min‐max)	Serum vitamin K first test (µg/L)	Reference range (min‐max)	Antibiotic courses per year	Sip feed per 24 h (ml)	Gastrostomy feed per 24 h (ml)
P1	22	F	30.89	17.36‐50.90	0.12	0.15‐1.55	2	150 ml	0
P2	60	M	58	17.36–50.91	0.68	0.15–1.56	2	0	0
P3	57	M	82.53	17.36–50.92	0.21	0.15–1.57	0	0	0
P4	74	F	25.19	17.36–50.93	<0.10	0.15–1.58	UK	0	0
P5	90	M	29.63	17.36–50.94	0.51	0.15–1.59	0	0	400
P6	91	M	1.22	0.00–0.20	0.29	0.15–1.60	2	0	0
P7	82	F	14.36	17.36–50.96	<0.10	0.15‐1.61	4	0	0
P8	114	F	Not done	17.36–50.97	0.45	0.15–1.62	1	0	800
P9	120	M	33.33	17.36–50.98	0.2	0.15–1.63	2	0	1000
P10	116	F	28.2	17.36–50.99	0.34	0.15–1.64	0	600	0
P11	115	F	43.85	17.36–50.100	0.12	0.15–1.65	0	0	No
P12	138	F	44	17.36–50.101	0.55	0.15–1.66	4	0	800 ml
P13	131	M	32.25	17.36–50.102	0.1	0.15–1.67	3	0	0
P14	159	F	30.23	17.36–50.103	0.17	0.15–1.68	3	0	200
P15	173	F	0.82	0.00–0.20	0.14	0.15–1.69	4	0	0
P16	180	F	25.09	17.36–50.105	0.74	0.15–1.70	UK	400	0

## 5 DISCUSSION

Our cohort of children and young adults with RDEB‐S had a high rate of vitamin K_1_ deficiency. These findings confirmed the suspicion that patients with RDEB‐S are at high risk for this deficiency due to their compromised oral intake, chronic malnutrition and likely secondary gut dysbiosis due to frequent antibiotic prescription and slow transit time.^15^, ^16^ In addition, patients with RDEB‐S can commonly display poor bone mineral density; suffering from fractures and osteopenia/osteoporosis.^3^, ^4^, ^5^, ^17^ It is well documented that patients with RDEB‐S are at high risk of both vitamin D deficiency and from the negative impact of chronic inflammation on bone mineral density^17^; however, it is as yet unknown whether chronic vitamin K deficiency may also play a contributing role in this chronic bone disease.

Antibiotic use in this cohort ranged from 0 to 4 courses prescribed (median = 3, IQR = 3) per year. The number of courses of antibiotics did not directly correlate with a higher risk of vitamin K deficiency. However, it is documented that changes in microbiome can be long‐term once a course of antibiotics has been taken, so it may not be dose‐dependent, rather, that antibiotics were given at all. However, where the number of antibiotic prescriptions were high, the 200 ml minimum of sip feed/enteral feed remained protective for deficiency; that is, antibiotic use may impact on risk of developing deficiency but the small daily dose provided by the nutritionally complete feed was enough to protect against deficiency. Further studies to elucidate how probiotics/prebiotics may improve the diversity of favourable bacteria in the microbiome of patients with RDEB‐S may play a future role in preventing vitamin K deficiency.

For patients in this study that were diagnosed with vitamin K deficiency, treatment was administered in two ways; if tablets were tolerated, menadiol diphosphate tablets have been prescribed; if tablets are not tolerated, ampules of phytomenadione were given orally (or via gastrostomy). As no treatment dose/guidelines exist for the treatment of vitamin K deficiency in children and young people with EB, the treatment dose was based cautiously at the lowest end of the recommended dose advised for treating conditions of paediatric vitamin K deficiency secondary to malabsorptive disorders. This resulted in a pharmaceutical dose of 5 mg every day for 12 weeks for children 1–10 years and 10 mg every day for 12 weeks for 12–17 year olds.^18^ Repeat samples in two of six patients revealed that this was sufficient to bring serum levels within the normal range and a prophylactic general multivitamin and mineral containing vitamin K has been advised thereafter.

The results indicate a protective effect of prescribed nutritionally complete sip/enteral feeds. All patients who had a minimum of 200 ml (content 5.9–11 µg/100 ml) of a nutritionally complete sip feed/enteral feed prescribed had sufficient levels of serum vitamin K_1_ and normal PIVKAII levels. Indicating that a minimum of a small daily dose of vitamin K (11.8–22 µg) was sufficient to maintain normal serum status of vitamin K_1_. This value is in line with current recommendations for dietary reference values for vitamin K in the UK: birth‐1 year requirements are estimated at 10 µg/day. Years 1–18 for boys range between 12.1 and 61.5 µg/day and for girls 1–18 years range from 11.5 to 55.6 µg.^19^

The age range of detected deficiency ranged from 22 to 180 months. The youngest patient in our cohort has a severe clinical presentation of RDEB‐S and displays vitamin K deficiency despite consumption of a long‐term high‐energy infant formula from 2 to 17 months of age that contained vitamin K, up until 5 months before testing. This illustrates that deficiency can develop quickly with high turn‐over in the liver, particularly where demands are high and dysbiosis is suspected. Data were not available on whether neonatal vitamin K doses had been given at birth, but would be an interesting future consideration for identifying risk factors.

A limitation of this study is the small sample size; a wider study looking at greater participant number of all subtypes of EB is now underway to see how the rate of deficiency compares in less severe subtypes too. A second limitation is the serum marker's specificity; although when used in combination with PIVKAII accuracy is improved, dietary intake will have an effect upon the serum K_1_ levels on the day. A proposed explanation as to why we have one patient who has normal serum K_1_ but perpetually raised PIVKAII levels is that their oral intake of vitamin K is high on analysis and may be adequate for immediate physiological needs. However, vitamin K stores are marginal and thus the hepatic ɣ‐carboxylation of factor II has been compromised. It remains unknown if it is clinically indicated to supplement this patient with vitamin K, however, ensuring adequate dietary intake and starting a daily multivitamin that contains a small amount of vitamin K would be prudent in this case.

Practice at our centre has now changed for high‐risk children with RDEB who are not taking at least 200 ml of a prescribable sip/enteral feed to ensure a complete vitamin and mineral supplement (containing vitamin K) is routinely advised to prevent vitamin K deficiency. Further analysis of bone mineral density studies, before and after detection and treatment of vitamin K deficiency, would be a valuable addition to the knowledge base of this subject. Future considerations will be to analyse the optimal length of time and dose of supplementation of vitamin K to treat the deficiency and how often this is required to maintain adequate stores. The appropriate and optimal compound within the vitamin K family to supplement with is also a future area of interest.

## CONFLICTS OF INTERESTS

The authors declare that there are no conflicts of interests.

## AUTHOR CONTRIBUTIONS

Natalie Yerlett conceived the presented idea and carried out all data collection and wrote all versions of the written report. Katie Holsgrove provided additional data collection. Anna Martinez supported all areas of the planning and writing up of the project. All authors read through the drafts of the manuscript with suggestions for improvements.
